# Railroading removal of gall bladder in laparoscopic cholecystectomy

**DOI:** 10.4103/0972-9941.25676

**Published:** 2006-03

**Authors:** V Golash, S Rahman

**Affiliations:** Departments of Surgery, Sultan Qaboos Hospital, P. O. Box No. 98, Salalah - 211, Sultanate of Oman; * Surgery, Al Ain General Hospital, Al-Ain, Abu Dhabi, United Arab Emirates

Laparoscopic cholecystectomy can be performed using instruments even smaller than 3 mm in diameter and with two, three or four ports, depending on the choice of the operating surgeon. In any of these techniques of laparoscopic cholecystectomy, the gall bladder is usually removed through the umbilical port. This requires repositioning of the instruments and reorientation.[1,2] Using the standard four port technique, the gall bladder was removed in 772 cases of laparoscopic cholecystectomy, through the umbilical port, without changing the position or the size of instruments, using the modification described below.[3] A tense and distended gall bladder was decompressed prior to removal and the fascial opening was enlarged for a large gall bladder or with a large stone. It is a safe, quick and efficient way of removing the gall bladder after laparoscopic cholecystectomy.

## Modification

Between June 1999 and September 2005, we recovered the gallbladder in 772 conventional laparoscopic cholecystectomies through the umbilical port, by using the technique described below. In 54 patients the gall bladder was acutely inflamed. The position of the ports and the patient were the same as for convention laparoscopic cholecystectomy, using three 5 mm ports and one 10 mm umbilical port. We routinely ligate the cystic duct and this positioning of ports is also ideally suited for ligation. The gall bladder was removed in all the cases of laparoscopic cholecystectomy through the 10 mm umbilical port, using the technique as described.

**Figure 1:**
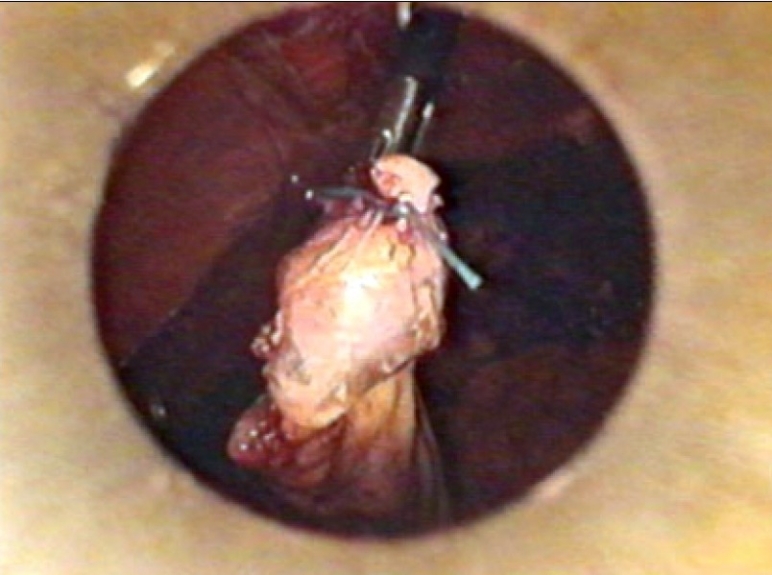
Gall bladder held on grasper in midclavicular

**Figure 2:**
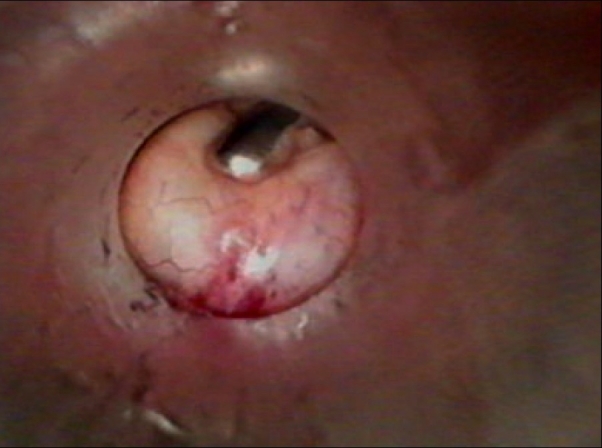
Gall bladder engaged in the 10 mm umbilical port

**Figure 3:**
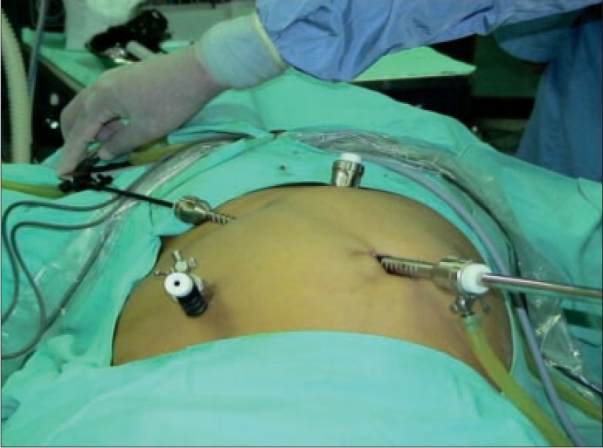
Railroading

**Figure 4:**
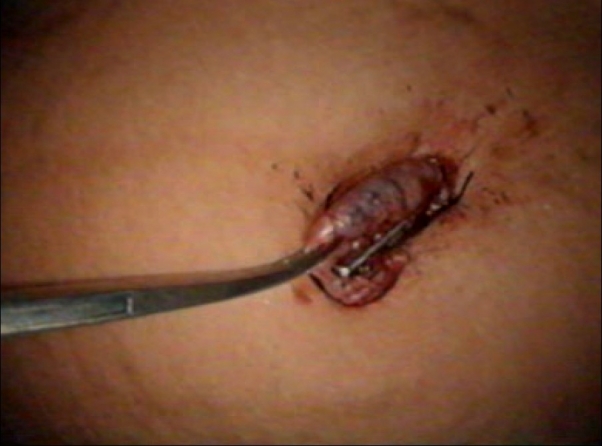
Gall bladder held on artery forceps & grasper released

The freed gall bladder was grasped at the cystic duct end by the grasper in the mid-clavicular port and was engaged inside the 10 mm umbilical port under direct vision [Figures 1 and 2]. The grasper in the mid-clavicular port and the telescope in the 10 mm ports, were kept in a straight line, to achieve this alignment [Figure 3]. By holding the gall bladder on the grasper, it was snugly accommodated further inside the port as comfortably as possible. The abdomen was deflated and the 10 mm umbilical port with the telescope was slowly withdrawn. Once the cystic duct end of the gall bladder with the grasper was visible outside, the gall bladder was held on the long artery forceps. The grasper was released, closed under vision and withdrawn [Figure 4]. The gall bladder was manipulated out slowly and removed. Compared to other techniques, the whole procedure was done under direct vision.[4] A similar technique was used for a large inflamed, edematous gallbladder, gall bladder with large stones and in case of accidental rupture of gall bladder, by placing the gall bladder in a custom made plastic bag and retrieving the bag by its long tail end at the umbilical port.

## Benefits

**Figure 5:**
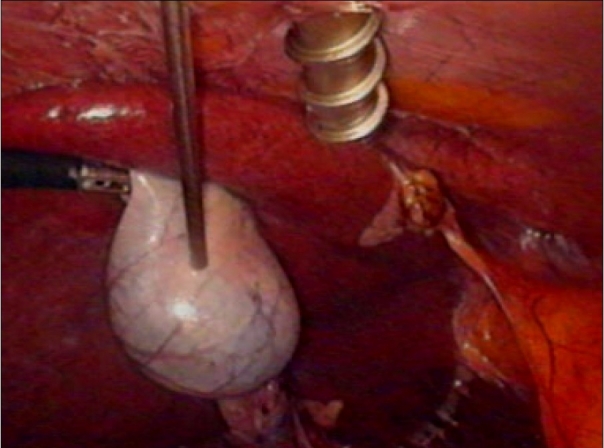
Aspiration of gall bladder by verres needle

In the technique described above, the removal of gall bladder through the umbilical port was simple, keeping the same arrangement of instruments and using only one 10 mm port. The decompression of a distended or a tense gall bladder was done by direct puncture and aspiration with the Verres needle percutaneously [Figure 5]. For a large gall bladder, or one with large stones, the umbilical port was enlarged further by slipping a long Kelly artery forceps by the side of the gall bladder for stretching the fascia, or dividing it longitudinally between its open jaws, if required.[5] There was no failure, no conversion to open and no complications. It is a safe and effective technique.
